# Lack of acclimatization to chronic hypoxia in humans in the Antarctica

**DOI:** 10.1038/s41598-017-18212-1

**Published:** 2017-12-22

**Authors:** Simone Porcelli, Mauro Marzorati, Beth Healey, Laura Terraneo, Alessandra Vezzoli, Silvia Della Bella, Roberto Dicasillati, Michele Samaja

**Affiliations:** 1 Institute of Molecular Bioimaging and Physiology, National Research Council, Segrate, (MI) Italy; 2 Biomedical Research, European Space Agency, Concordia, Antarctica; 3 Department of Health Science, University of Milan, Milan, (MI) Italy; 4 Department of Medical Biotechnologies and Translational Medicine, University of Milan, Milan, (MI) Italy; 5 ASST Santi Paolo e Carlo, Milan, (MI) Italy; 6 Unit of Clinical and Experimental Immunology, Humanitas Clinical and Research Center, Rozzano, Milan Italy

## Abstract

The study was carried out at Concordia Station (Antarctic Plateau). The aim was to investigate the respiratory and haematological responses to hypoxia in healthy subjects living at constant altitude. Thirteen men and women (34.1 ± 3.1 years) were exposed for 10 months to hypobaric hypoxia (oxygen level equivalent to 3800 m asl). These unique conditions enable a greater accuracy of monitoring human responses to chronic hypoxia than can be achieved elsewhere. Blood haemoglobin and erythropoietin concentrations were determined at sea level (Pre), and after 3, 7, 20, 90 and 300 days at altitude. Blood gas analysis, base excess and arterial oxygen saturation were measured at Pre, and after 150 and 300 days at altitude. Erythropoietin returned quickly to baseline level after a transient increase in the first days. Blood haemoglobin concentration started increasing at day 7 and remained markedly higher for the entire duration of the mission. At day 150 the blood carbon dioxide partial pressure was markedly reduced, and consequently blood pH remained higher at negative base excess until day 300. The arterial oxygen saturation remained lower than Pre throughout. In conclusion, humans display little capacity of hypoxia acclimatization even after ten months of constant exposure to low oxygen partial pressure.

## Introduction

Despite the increasing number of people who are exposed to moderate or high altitude, sojourning at altitude can still represent a pathophysiological challenge due to systemic hypoxia, i.e., low oxygen supply with respect to the body’s need. Although humans can acclimatize, at least partially, to moderate and brief oxygen shortages, hypoxia remains a potentially lethal situation and has significant consequences, both in terms of lives lost and social costs for care and rehabilitation. The condition of hypoxia may be further aggravated by maladaptive patterns leading to pathological conditions, though this issue has not yet been examined sufficiently to develop safe and adequate therapeutic countermeasures.

Long-term acclimatization to hypoxia of sea-level dwellers involves metabolic, respiratory, circulatory and genetic mechanisms^1^, which may or may not be fully engaged in order to ensure survival and ability to perform work at altitude. Among these, the mechanisms relating to respiratory acclimatization are particularly suitable to monitor hypoxia acclimatization. Yet the comprehension of these mechanisms requires the availability of experimental models wherein altitude hypoxia represents the only major variable, with exclusion of poorly controllable factors such as strenuous exercise, psychological stress, excessive temperature fluctuations, altitude changes, irregular feeding and liquid consumption. As such, Concordia Station in Antarctica is an ideal place to study human acclimatization to hypoxia. The station is located at 3233 m asl, but high latitude further reduces the local barometric pressure, causing the amount of oxygen in breathed air to correspond to an altitude of approximately 3800 m in more moderate latitudes. These conditions are generally far from dangerous in terms of the exposure to hypoxia, and healthy humans are predicted to adapt with little risk of altitude illness. Hence, subjects who overwinter in the Concordia facility are exposed to “moderate hypoxia” for up to 10 months without any change in altitude, thereby providing the opportunity to get an insight into the effects of moderate hypoxia in the absence of disturbing factors.

## Methods

### Subjects

Thirteen healthy volunteers (average age 34.1 ± 3.1, range 24–56 years) from France and Italy participated in this study during their stay at Concordia Station. They were recruited through advertisements and announcements and prior to their enrolment, participants underwent rigorous medical and psychological testing. Subjects were then asked to sign a declaration in which they were informed of the risks of the study and agreed to its terms. The study was approved by both the Commission for Research Bioethics of the Italian CNR and the Ethical Committee of the San Paolo Hospital in Milan. All procedures and methods were performed in accordance with the relevant international guidelines and regulations in order to reduce physical discomfort of the subjects. This study was conducted within the framework of the European Space Agency’s (ESA) Life Science campaign at Concordia Station.

### Study design

The data reported in this study were obtained during the overwinter period 2014–2015. Baseline measurements (Pre) were performed before the beginning of the mission at the European Space Agency Centre in Cologne (Germany, 91 m asl). During the mission, blood venous samples for haematological measurements were obtained 3, 7, 20, 90 and 300 days after arrival at Concordia Station. In addition, capillary fingertip samples for blood gas determinations were obtained on day 150 and 300. Concordia Station is a French-Italian research facility located at Dome C on the Antarctic Plateau at 3233 m asl. Although the average air temperature, humidity and wind speed outside the Station were −58 ± 9 °C, 41 ± 10% and 2.8 m/s respectively, the temperature inside, where the team spent virtually all of their time, was permanently 22 ± 2 °C. The mean barometric pressure during the winter (637 hPa or 478 mmHg) corresponds to an altitude of approximately 3800 m asl in more moderate latitudes, a value slightly lower than reported elsewhere^2^. Indeed, relatively large fluctuations in monthly barometric pressures have been recorded in Antarctica, with lower values, and hence higher equivalent altitudes, during the winter months^3^. Interestingly, accurate measurements have shown that the oxygen fraction in Antarctic air is lower than that in the rest of the world, ranging from 20.82 to 20.90%^4^. Thus, we assume that the actual equivalent altitude of Concordia is 3800 m asl.

The incidence of high altitude illness tended to be higher than in other locations at the same altitude^5^, and as such, all subjects were examined weekly by a staff physician for assessment of the Acute Mountain Sickness (AMS) score according to the Lake Louise symptom scale^6^.

### Haematological measurements

Two blood samples were collected from the antecubital vein in heparinized vacuum tubes (4 ml each). One tube was frozen immediately, while the other was quickly centrifuged (1000 g for 10 min) and plasma placed in a separate tube and frozen. At the end of the 10-month stay, frozen samples were transferred to Milan for the analysis of total haemoglobin (Drabkin’s method) and plasma level of erythropoietin (EPO, sandwich enzyme-linked immunoassay, Quantikine), respectively. For EPO quantification, optical density was measured in a Quant microplate reader (Bio-Tek instruments, Winooski, VT, USA, CV 3.1%, sensitivity 0.2 mU/mL).

### Blood gas measurements

Capillary blood samples were obtained from the palm-up surface of the distal segment of the middle finger. Blood was collected into heparinized 100 µL capillaries and immediately analysed for pH, PCO_2_ and Base Excess (GEM Premier 3000, Instrumentation Laboratory, USA). The arterial oxygen saturation (SaO_2_) was measured immediately after by fingertip pulse oximetry (Nonin Medical Inc., Plymouth, Minnesota, USA).

### Statistical analysis

Data are reported as average, median, range and 10^th^–90^th^ percentile. One-way ANOVA analysis was followed by the Dunnett’s multiple comparison test to address significant (P < 0.05, two-tailed) deviations from baseline (Pre) values.

### Data availability

The data obtained in anonymized subjects, the full dataset, statistical details are available from the corresponding author (MM).

## Results

The environmental conditions at Concordia Station are predicted to cause some degree of emotional stress due to long isolation, but all subjects were in good health for the whole duration of the study and never experienced relevant clinical signs of high altitude illness. Routine appropriate standards ensured highest reliability of blood gas analyses.

**Figure 1:**
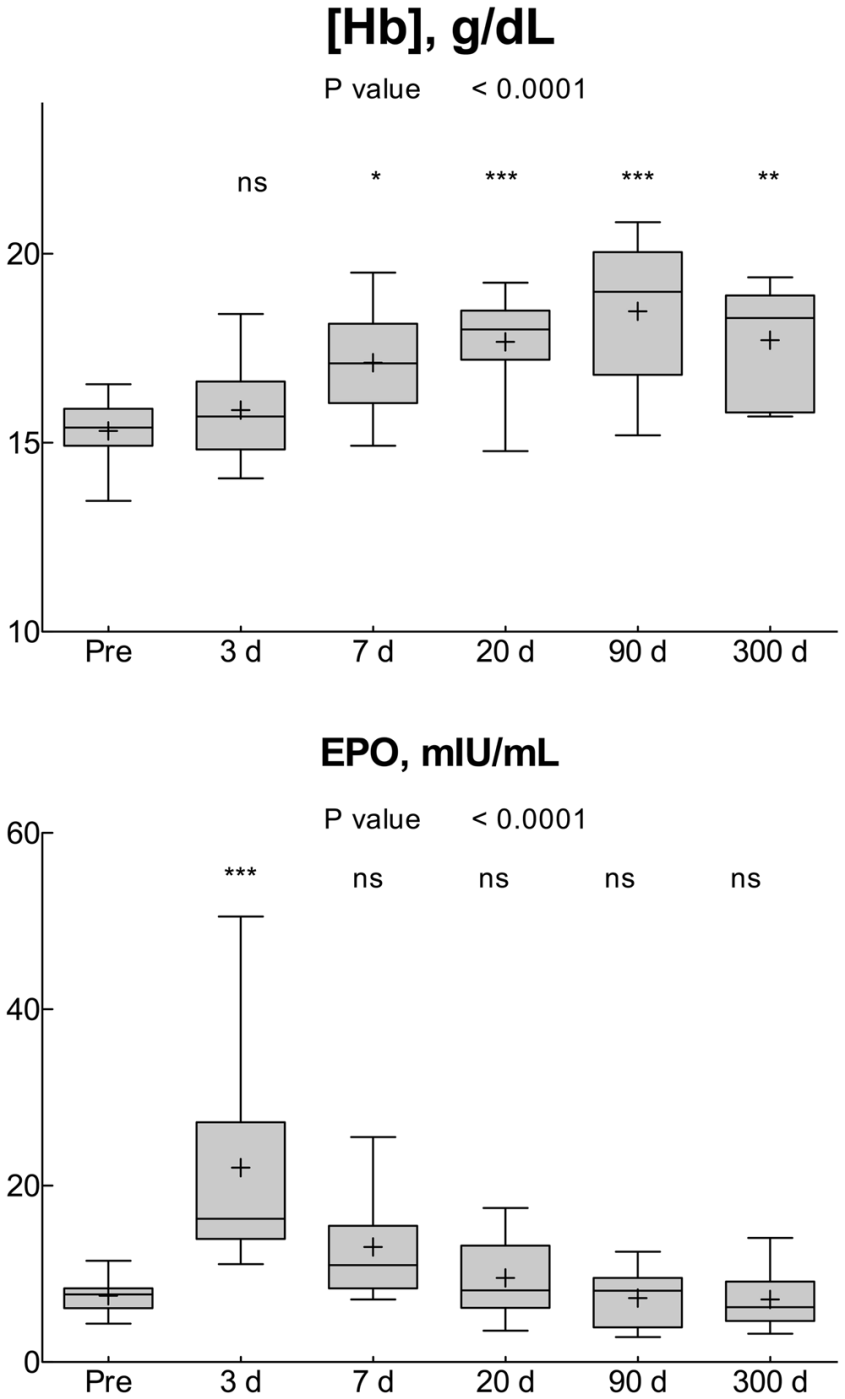
Whisker plots representing the 10–90% percentile (upper and lower frames of the box) and range (bars) for the variables measured in this study. The plus sign and the horizontal bars within each whisker represent the mean and the median, respectively. The P values of the one-way ANOVA are also shown. The symbols *^,^ ** and *** represent P < 0.05, P < 0.005 and P < 0.0005, respectively, vs Pre (Dunnett’s multiple comparison test), ns = not significant (P > 0.05), n = 13 for all points.

Blood Hb concentration began increasing since day 7 after the beginning of altitude exposure (Fig. 1), and remained higher than Pre, without signs of normalization until day 300. By contrast, plasma EPO peaked at day 3 (Fig. 1), but quickly normalized by day 7 and remained virtually indistinguishable from Pre until the end of the study.

**Figure 2:**
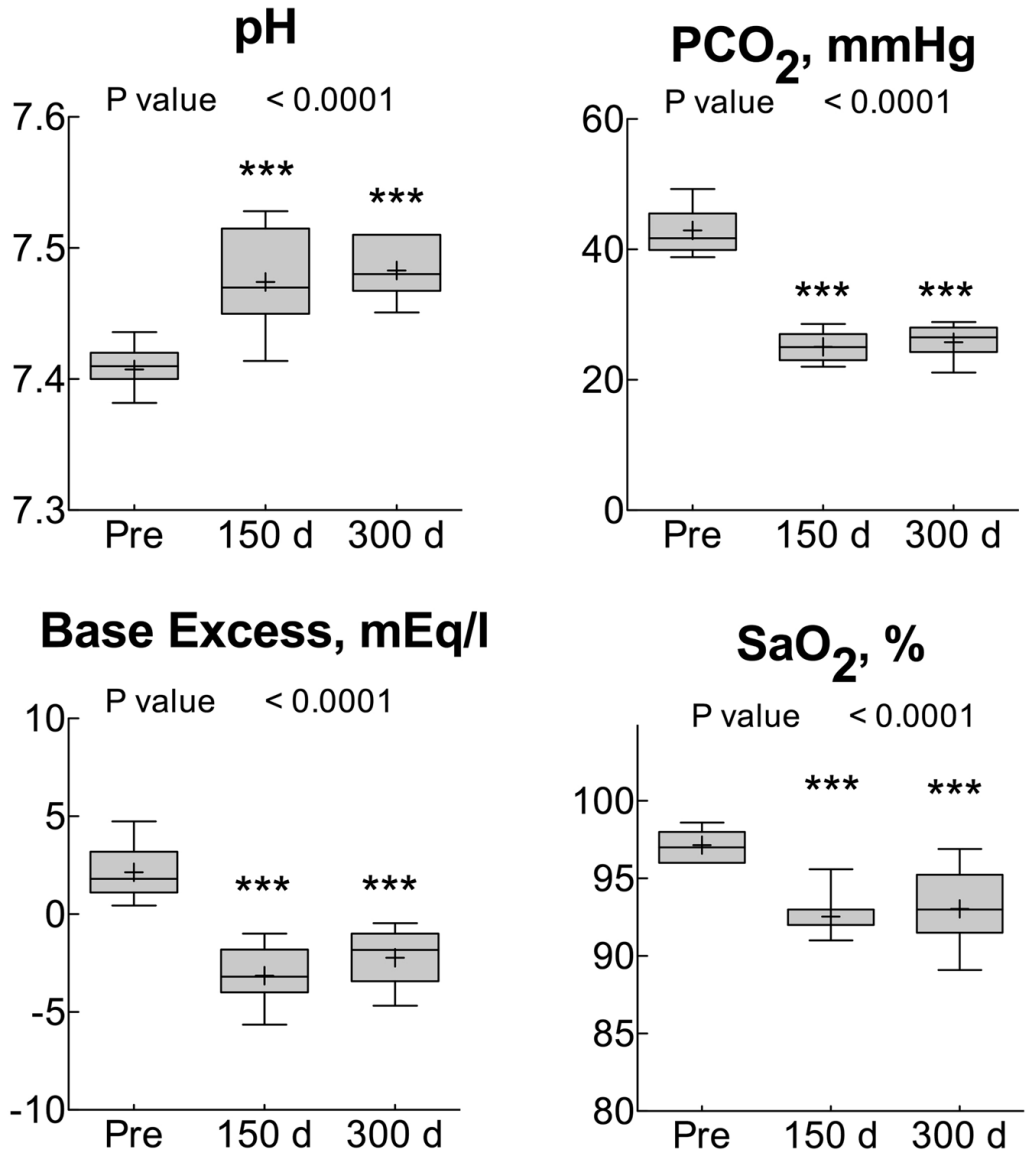
Whisker plots representing the 10–90% percentile (upper and lower frames of the box) and range (bars) for the variables measured in this study. The plus sign and the horizontal bars within each whisker represent the mean and the median, respectively. The P values of the one-way ANOVA are also shown. The symbols ** and *** represent P < 0.005 and P < 0.0005, respectively, vs Pre (Dunnett’s multiple comparison test), ns = not significant (P > 0.05), n = 13 for all points.

Figure 2 shows blood gas analyses performed at sea level (Pre) and at day 150 and 300 days after arrival at Concordia Station. SaO_2_ remained lower than Pre throughout. Venous PCO_2_ decreased considerably and remained lower than Pre for the whole duration of the altitude exposure without showing any normalization trend. Consequently, pH remained higher, and the Base Excess was lower than Pre up to day 300.

## Discussion

This study shows a significant reduction in blood PCO_2_, and consequently an increase in blood pH at negative Base Excess, in healthy subjects residing at altitude for 300 days, along with a transient increase in the first days of EPO plasma level and a persisting higher blood Hb concentration. Thus, the results of the present study suggest that humans display little capacity for hypoxia acclimatization even after ten months of constant exposure to reduced PO_2_.

When humans are exposed to hypoxia, several physiological changes occur in the muscular, respiratory, cerebral, cardiovascular and hormonal systems. Hypoxia is known to increase alveolar ventilation through stimulation of chemoreceptors, with excessive CO_2_ washout from the lungs, reduced arterial PCO_2_ and increased blood pH. Whereas, at sea level, pH fluctuates within a narrow range - around 7.38 - at altitude, the mechanisms that titrate pH back to normal values are less efficient; indeed, the renal compensation is too slow to cope with continuous CO_2_ washout in the lungs. Consequently, the blood gas analysis gives a unique insight into the process of acclimatization to hypoxia because, although increasing the rate of breathing represents a countermeasure to oxygen shortage, it also represents a cost in depleting the body of CO_2_ reserves and impairing the control of blood acidity. Previous data obtained after 7–10 days at 6,450 m shows that arterial pH remains 7.496 ± 0.006 and Base Excess −6 mEq/l, whereas a Base Excess of −10 to −9 mEq/l would be necessary for full compensation^7,8^. These changes might represent a protective mechanism because they might improve oxygen binding to Hb, but blood alkalosis is not compatible with normal body function. In fact, less acclimatized subjects are characterized by more marked alkalosis with respect to better acclimatized subjects^8^. Furthermore, acetazolamide, a drug that fights AMS, tends to lower blood pH by inhibiting carbonic anhydrase^9^. However, such observations were obtained under conditions of short, albeit severe hypoxia, though it remains to be clarified whether longer exposure to altitude, even moderate, can eventually normalize blood alkalosis; pointing to a phenotype in which hypoxia “adaptation” might ever be possible in humans. In this study, blood gas analysis data shows a significant reduction of PCO_2_, increased pH and negative Base Excess even after 10 months at altitude. These data were obtained in healthy subjects sojourning at Concordia Station, a unique location at altitude which prevents interference of factors like altitude changes, indiscriminate exercise, environment stress, and cold. Thus, the present study points out that respiratory acclimatization to altitude in the absence of confounding factors is still an unaccomplished goal for altitude exposures of up to 300 days.

Since the sixties of the past century, it is well known that a long sojourn (more than eight months) at high altitude leads to polycythaemia and decreased PCO_2_^10^. However, these data were obtained during mountaineering expeditions possibly characterized by confounding factors such as strenuous exercise, psychological stress, altitude changes, irregular feeding and liquid consumption as it happened, for example, in the historical Silver Hut expedition in 1960–1961 at 5800 m^11^. Very few studies have been conducted in lowlanders living for months/years at constant altitude under reasonable environment stress conditions. In general, living at moderate altitude is inversely associated with the risk of developing overweight/obesity and metabolic syndrome^12,13^ and may have a protective effect on ischemic heart disease, stroke, COPD and cancer^14^. Short (fourteen days at 2650 m) studies of obese patients caused PCO_2_ to fall from 37.0 ± 0.9 to 29.1 ± 0.8 mmHg (no pH data) and SaO_2_ from 95.2 ± 0.3 to 88.5 ± 1.8% with considerable weight loss^15^. Also, non-overweight subjects at 1700m for 19 days did not develop a measurable increase in blood Hb concentration but EPO peaked around two-fold after 15–17 h at altitude followed by slow return to baseline^16^. A similar time course of plasma EPO changes has been documented at altitudes >3600 m in the Andes^17^ al well as by several groups (reviewed in ref.^18^) and is in agreement with the results of the present study, although other factors such as dehydration due to dry atmosphere may play a role.

Lack of normalization of Base Excess after a 300-day exposure to altitude is in contrast with a previous study performed in ten Han immigrants, born at sea level but residing at an altitude of 3600 m for 25.6 ± 9.1 years^19^. In that study, arterial blood pH averaged 7.419 at PCO_2_ = 27.3 ± 4.5 (SD) mmHg, thus indicating full normalization of blood pH at a cost of Base Excess = −6.6 mEq/L, much less than that found in the present study. Nevertheless, present data are in agreement with another study in Han immigrants to the Qinghai-Tibetan plateau^20^ revealing that SaO_2_ remained unchanged between those who migrated before 1987 and after 1988 (better preserved), whereas blood Hb concentration and other physical parameters, such as stature and weight changed considerably. Overall, these observations might address the notion that at altitude the subject aims to prevent excessive decrease in SaO_2_ at the cost of higher Hb concentration and lower Base Excess. Indeed, it was suggested that hematopoietic response to moderate altitude is expected to occur in mammalian species that are not genetically adapted to high altitude^21^. In the present study, venous blood data were used instead of arterial or arterialized blood to assess Base Excess and it may be considered an apparent limitation. However, several comparative studies (reviewed in ref.^22^) have shown satisfactory agreement between resting arterial and venous blood pH, PCO_2_ and Base Excess, especially for subjects who are not in shock, as in this study. This agreement is confirmed in another study that included trauma patients^23^, but has not been demonstrated with mechanically ventilated patients^24^. As the PO_2_ is expected to diverge significantly in arterial and venous samples, this variable was not included in this study and was replaced by arterial O_2_ saturation.

It might be matter of discussion whether establishing a steady set point after 300 days at altitude represent a form of successful acclimatization. Indeed, it is questionable if higher blood hemoglobin and alkalosis represent beneficial ways to adapt to environment hypoxia because of the associated potentially disturbing effects on blood viscosity and possible damage to kidney and cerebral tissues. Apart from the biological considerations linked to the failure of humans to fully adapt even to moderate hypoxia, which appears to be especially true for permanent residents^25^, it is a matter of further investigation whether sustained blood alkalosis, as that found in this study, might turn out to be harmful. This issue may become particularly relevant when planning the optimal environmental conditions during long spacecraft missions where the oxygen fraction in the inspired atmosphere and the pressure may be altered. Data gathered in Concordia Station suggests that cerebral function may indeed become compromised, as supported by several observations in which sustained hypoxia exposure induces brain damage at both the experimental and clinical levels. For example, chronic 2-week hypoxia equivalent to 5000 m increases neuron apoptosis through down-regulation of the NO/cGMP pathway^26^. In addition, the effect of chronic hypoxia on cognitive impairment is now recognized^27–30^, and oxygen supplementation in high-altitude schools has been proposed as a valid countermeasure to prevent interference with the learning processes^31^.

In conclusion, it is well known that hypoxia affects several physiological functions and the overall quality of life. However, it is not clear whether prolonged exposure to moderate hypoxia can eventually be tolerated because of the lack of suitable experimental models in which hypoxia represents the only major variable. This study, conducted in a relatively controlled environment that excludes many of the typical confounding factors such as strenuous exercise, psychological stress, excessive temperature fluctuations, altitude changes, irregular feeding and fluid consumption, appears to indicate that humans lack complete acclimatization to moderate altitude even after 10 months according to persisting respiratory and haematological changes. Further studies should evaluate long-term effects of lack of acclimatization to hypoxia on cerebral and renal functions.
