# Author Correction: Lack of acclimatization to chronic hypoxia in humans in the Antarctica

**DOI:** 10.1038/s41598-018-24804-2

**Published:** 2018-05-01

**Authors:** Simone Porcelli, Mauro Marzorati, Beth Healey, Laura Terraneo, Alessandra Vezzoli, Silvia Della Bella, Roberto Dicasillati, Michele Samaja

**Affiliations:** 1 Institute of Molecular Bioimaging and Physiology, National Research Council, Segrate, (MI) Italy; 2 Biomedical Research, European Space Agency, Concordia, Antarctica; 3 Department of Health Science, University of Milan, Milan, (MI) Italy; 4 Department of Medical Biotechnologies and Translational Medicine, University of Milan, Milan, (MI) Italy; 5 ASST Santi Paolo e Carlo, Milan, (MI) Italy; 6 Unit of Clinical and Experimental Immunology, Humanitas Clinical and Research Center, Rozzano, Milan Italy

Correction to: *Scientific Reports* 10.1038/s41598-017-18212-1, published online 22 December 2017

The original version of this Article omitted an affiliation for Silvia Della Bella. The correct affiliations are listed below:

Department of Medical Biotechnologies and Translational Medicine, University of Milan, Milan, (MI), Italy.

Unit of Clinical and Experimental Immunology, Humanitas Clinical and Research Center, Rozzano, Milan, Italy.

This has now been corrected in the HTML and PDF versions of this Article.

